# PEGylated Carbon Nanotubes Decorated with Silver Nanoparticles: Fabrication, Cell Cytotoxicity and Application in Photo Thermal Therapy

**DOI:** 10.22037/ijpr.2019.112339.13697

**Published:** 2021

**Authors:** Mohammad Ali Behnam, Farzin Emami, Zahra Sobhani

**Affiliations:** a *Nano Opto-Electronic Research Center, Department of Electrical and Electronics Engineering, Shiraz University of Technology, Shiraz, Iran. *; b *Department of Quality Control, Faculty of Pharmacy, Shiraz University of Medical Sciences, Shiraz, Iran.*

**Keywords:** Photothermal therapy (PTT), O-CNTs, O-CNT/Ag-PEG, Laser therapy, Silver nanoparticles

## Abstract

A new technique for cancer therapy is Photo Thermal Therapy (PTT). In the PTT technique, photon energy is converted into heat via various operations to destroy malignant tumors. Carbon nanotubes (CNTs) have good optical absorption in the near-infrared (NIR) spectrum and could transform optical energy into heat to induce hyperthermia in the PTT method. In this study, CNTs were firstly oxidized (O-CNT) and then decorated with silver nanoparticles (Ag NPs). Polyethylene glycol (PEG) was utilized for wrapping the surface of CNTs (O-CNT/Ag-PEG). Coating of CNTs with Ag NPs and PEG was confirmed by XRD, FESEM, and TEM techniques. Results demonstrated that noble metal could increase optical absorption of CNTs and concurrently improve the efficacy of the PTT technique. Cell cytotoxicity study showed that O-CNT/Ag NPs were less cytotoxic than O-CNTs, and O-CNT/Ag-PEG had the lowest toxicity against HeLa, HepG2, and PC3 human cell lines. The efficacy of O-CNT/Ag-PEG NPs in destroying malignant melanoma tumors was evaluated through the PTT technique. A continuous wave NIR laser diode (λ = 808 nm, P = 2 W, and I = 2 W/cm^2^) irradiated the tumor sites for 8 min once in the period of the treatment. The tumors in cases receiving O-CNT/Ag-PEG were shrunk efficiently compared to laser treatment ones. Results of *in-vivo *studies demonstrated that O-CNT/Ag-PEG was a puissant candidate in extirpating malignant tumors in PTT method.

## Introduction

Cancer still remains the worst harmful disease in the world. Cancer is the second leading cause of death in the United States. According to the American Cancer Society in 2019, in the US population, approximately 1.7 million new cancer cases will be recognized and more than 4800 new cases are reported daily. Moreover, it is predicted that around 1700 cancer death will occur each day in the US in 2019. Besides, cancer cost in the US was expected to be $173 billion for the year 2020 (1, 2). Surgery, chemotherapy, and radiotherapy are the main strategies for cancer treatment. All these methods have side effects and specific limitations for cancer treatment. Nanotechnology plays an important role in the diagnosis and treatment of cancer. Prospective applications of nanoparticles (NPs) include imaging, drug delivery, anti-cancer activity, and photothermal therapy (1, 3 and 4).

A new technique which changes the methods of cancer treatment is photothermal therapy (PTT). PTT method includes engineering, pharmaceutics, material sciences, optics, and physics with cancer biology (5).

PTT is an uncomplicated method of cancer therapy in which near infrared (NIR) light raises the temperature of tumor site and terminates the cancer cells. Localized NPs could be provoked by a laser diode and eventually induce heat in the range of 40-47 ◦C in the tumor sites and extirpate cancer cells (6). NPs such as multi-walled carbon nanotubes (MWCNTs) (5), graphene (7), gold NPs (8–11), and titanium dioxide (12) are used to convert NIR beam into heat (13).

Metal NPs have numerous utilization in photonics, electronics, imaging, and chemical sensing (6). Metal NPs, especially noble metal NPs, could interact with the different spectrum of light. These characteristics of mentioned NPs could provide various applications in Raman scattering, radiative rate enhancement, optical biosensor, cancer treatment, and biological applications (5, 6). Plasmonic (noble metal) NPs have been distinguished from other NPs by their unique surface plasmon resonance (SPR). This unique characteristic enhances radiative and non-radiative features of plasmonic NPs completely (6, 12). Plasmonic NPs, like silver and gold, could be modified to absorb light at a particular wavelength in the NIR spectrum. The optical absorption of NPs is proportioned to the size, structure, and material nature of NPs (14).

Many diverse effects have been reported for silver (Ag) NPs such as antimicrobial, antioxidant, anti-inflammatory, and anti-cancer activity (15–19). To date, photothermal activity of Ag NPs is reported by several studies (20–22). The combination of Ag NPs with other materials can create nanocomposites in which the photothermal property is enhanced (20, 21). 

Carbon nanotubes (CNTs) have unique properties such as high aspect ratio, ultra-light weight, high mechanical strength, high optical absorption, and high electrical and thermal conductivity which are special for nanotechnology, optoelectronics, and biomedical engineering (5, 23). CNTs have developed greatly in drug delivery systems and could operate as vehicles for drugs, antigens, and imaging agents (24). The optical absorbance spectrum of CNTs assures an exceptional property over plasmonically heated nanomaterials dependent on the size and structure of CNTs. Some pharmacokinetic studies have shown that the combination of CNTs and noble metals could enhance the application of CNTs in both drug delivery system and PTT technique (5, 6).

Heat generation based on laser excitation of NPs localized in the tumors could raise cancerous tissues’ temperature and extirpate tumors by making physical disruption, such as protein denaturation and membrane lysis, and finally cause necrosis and apoptosis in tumor sites (25). NIR light (700 nm-1100 nm) could efficiently enter the body because biological systems largely lack chromophores to absorb this light spectrum. NIR beam is more transmissive through the body, and is rarely weakened by biological systems (5, 12). Therefore, decorating CNTs with noble metals could enhance the NIR absorption of CNTs and improve their beam energy conversion in the PTT technique.

To improve the dispersibility of NPs in aqueous media, various hydrophilic polymers could be utilized. Functionalization of NPs with polyethylene glycol (PEG) moieties (PEGylation) has been used to improve the biocompatibility, solubility, and cell membrane penetration of the moieties (5, 6 and 12).

In this research, we prepared CNTs decorated with Ag NPs, firstly and improved their aqueous dispersibility of these NPs with PEG. After *in-vitro *study and characterization of these NPs, we assessed their efficacy in PTT of melanoma cancer *in-vivo*. 

## Experimental

***Materials***

Purified MWCNTs (number of walls: 5-15, outer diameter: 5-20 nm, length: 1-5 μm) and PEG_1000_ were purchased from Plasmachem (Berlin, Germany) and Sigma-Aldrich (St Louis, MO, USA), respectively. AgNO_3_ was purchased from Merck Co. (Germany).

***Preparation of O-CNT-PEG and O-CNT/Ag-PEG***

To wrap CNTs with PEG_1000_, the CNTs were oxidized at first. Two grams of MWCNTs were sonicated with 40 mL nitric acid and sulfuric acid solution (1: 3 v/v) for 45 min. Then the solution was refluxed for 22 h at 110 ◦C. The solution was diluted with 2 L of deionized water, then filtered and washed till the pH adjusted to approximately 6. The filtrate was dried in an oven at 45 ˚C (5, 6). The oxidized-CNTs (O-CNTs) were prepared for the next steps.

Thirty milligrams of O-CNTs was added to 30 mL of deionized water containing 300 mg of PEG_1000 _(5). This mixture was ultra-sonicated for 12 min and stirred overnight to wrap hydrophilic polymer around the O-CNTs. The resulting solution was centrifuged at 4600 rpm for 15 min to remove aggregates, and the supernatant containing O-CNT-PEG_1000_ was saved. Free PEG was removed by dialysis (5).

For preparation of O-CNT decorated with Ag NPs, 100 mg O-CNTs was dispersed in 40 mL deionized water by 45 min ultra-sonication. Forty milliliters aqueous solution of 0.1 M AgNO_3_ was added dropwise to the resulting solution. The pH of suspension was adjusted to approximately 6 by adding 0.1 M NaOH solution, and it was ultra-sonicated another time for 50 min. Ultimately, the resultant O-CNT/Ag NPs were washed ten times with deionized water and were centrifuged to be removed from the solution (6). To enhance the dispersibility of O-CNT/Ag NPs in an aqueous solution, a layer of PEG_1000_ was coated on them. Fifty milligrams of O-CNT/Ag NPs were suspended in 50 mL of deionized water containing 500 mg of PEG_1000_. The suspension was ultra-sonicated for 12 min and stirred at room temperature overnight to allow for wrapping hydrophilic polymer around O-CNT/Ag NPs. The resulting solution was centrifuged at 4600 rpm for 15 min to remove aggregates, and the supernatant containing O-CNT/Ag-PEG was saved. Free PEG was removed by dialysis (5).

***Characterization of O-CNT-PEG and O-CNT/Ag-PEG***

The phase composition of O-CNT/Ag NPs was determined by X-ray diffraction (Bruker, XRD, Germany) analysis.

Field emission scanning electron microscopic (FESEM) analyses were accomplished (TESCAN MIRA 3-XMU, FESEM; Brno, Czech Republic), and also microscopic images were taken by a transmission electron microscope (Philips Electron Optics, TEM, The Netherlands). UV-Vis light absorption spectrum of O-CNT-PEG and O-CNT/Ag-PEG was analyzed (PG instruments Ltd., T80^+^ UV-Vis spectrophotometer, Lutterworth, UK) to determine the best optical absorption wavelength for laser incitation. 

*Cytotoxicity assay for functionalized CNTs*

The cytotoxicity of MWCNT, O-CNT, O-CNT/Ag, and O-CNT/Ag-PEG_1000_ was evaluated by standard dimethylthiazole-tetrazolium (MTT) assay. Three human cell lines, human cervical cancer cells (HeLa), human hepatocellular carcinoma cell line (HepG2), and human prostate cancer cell line (PC3), were purchased from the National Cell Bank of Pasteur Institute (Tehran, Iran). All human cell lines were cultured in RPMI-1640 medium (Shellmax, China) supplemented with 1% penicillin-streptomycin (Invitrogen, USA) and 10% fetal bovine serum (Shellmax, China) at 37 °C in a humidified incubator with 7% CO_2_. Cells in the exponential growth phase were seeded in 96-well plates at a density of 1 × 10^4^ viable cells/well. After 24 h incubation, the mentioned cells were faced with different concentrations of MWCNT, O-CNT, O-CNT/Ag and O-CNT/Ag-PEG_1000_. Twenty-four hours later, 20 μL of MTT (5 mg/mL) and 100 μL of medium were added. The plates were incubated for 4-5 h. The formazan crystals were dissolved in 120 µL of dimethyl sulfoxide. After dye solubilization, an ELISA reader recorded the plates at 570 nm against 690 nm. The cell viability was assessed by estimating the reduction of values from a dimethyl sulfoxide control, and the values were the means of three different experiments.

***Tumor Induction***

B16/F10, a metastatic murine melanoma cell line (NCBI C540), was bought from the National Cell Bank of Pasteur Institute of Iran, Tehran, Iran. It was cultured in RPMI 1640 medium, under 7% CO_2_ at 37 ◦C. After that, it was supplemented with 10% fetal bovine serum, 100 IU/mL of penicillin and 100 µg/mL streptomycin.

For tumor induction, inbred female C57BL/6J mice-weighing 20-25 g with ages of 4-7 weeks- were gathered. Murine melanoma cells at a density of 6 × 10^5^ were suspended in 150 µL culture medium and were injected hypodermically. This project was accomplished in the Center of Experimental and Comparative Medicine, Shiraz University of Medical Sciences, Shiraz, Iran and was admitted by the Ethical Committee of Shiraz University of Medical Sciences.

Selection, procedures, and euthanizing of mice were accomplished according to the guidelines of Animal Care Committee of Iran Veterinary Organization. Experiments were performed under aseptic conditions and the protocol of anesthesia, postoperative cares and surgical procedures were the same for all mice.

***Hyperthermia therapy of tumors***

The efficacy of cancer therapy employing O-CNT-PEG and O-CNT/Ag-PEG with a laser diode was assessed by monitoring the size of tumors inoculated in mice and histopathological examination. After fifteen days of melanoma cells injection, tumors were grown sufficiently (approximately 1 cm^3^) to begin PTT. The mice were grouped randomly into four groups (n = 5) and anesthetized by a combination of Ketamine 10% (100 mg/kg) and Xylazine 2% (10 mg/kg). After relaxation, the hair around the tumor was shaved and the skin was cleansed. Tumor sizes were measured using a caliper and assessed with an ultrasonography machine (Ultrasonix SonixOP; Burnaby, BC, Canada) before the treatment and four days after the treatment. The tumor size was calculated with the following Equation:

Tumor volume = (L/2) ×W^2^ (mm^3^) (26)

L and W indicate the length and width of the tumor, respectively.

The cancerous mice received treatment as follows: 

In Group 1 (CNT): O-CNT-PEG (1 mg/mL) was injected into the tumor at a dose of 150 µL/cm^3^ (tumor volume).

In Group 2 (CNT/Ag): O-CNT/Ag-PEG (1 mg/mL) was injected into the tumor at a dose of 150 µL/cm^3^ (tumor volume).

In Group 3 (Laser therapy): laser therapy was done without injection of any NPs.

Group 4 (Control): it did not receive any treatment.

Tumor area in groups 1, 2, and 3 was stimulated with an 808 nm continuous wave (CW) NIR laser diode with the intensity of 2 W/cm^2^, and spot size of 1 cm^2 ^for 8 min (5, 13 and 27). After the period of the treatment, all mice were euthanized, the tumor size was estimated, and the mass was excised for histopathological examination.

***Histopathological examination***

The excised masses were sent for histopathologic evaluation. The specimens were processed, and then formalin-fixed paraffin-embedded (FFPE) blocks were built and slides were stained with Hematoxylin and Eosin (H and E) technique. The specimens were assessed grossly and sampled for microscopy evaluation.

***Statistical analysis***

Data are shown as mean ± standard deviation (SD). Significant differences in the values were statistically assessed by Paired-Sample *t*-test in each group. Multiple comparisons at multiple time points were evaluated by ANOVA with Repeated Measures. The statistical analyses were accomplished by SPSS^®^ statistical software, version 20.0 (SPSS Inc., Chicago, IL, USA). A *P*-value of less than 0.05 was regarded as significant.

## Results

*Schematic figure*

**Figure 1:**
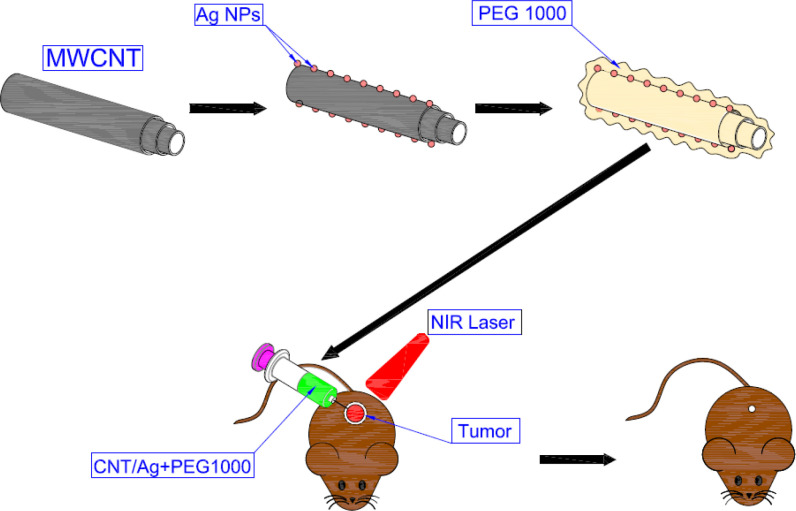
A schematic representation of the procedures and mechanism of PTT

Figure 1 shows a schematic representation of the procedures and mechanisms of PTT in cancer treatment based on functionalized CNTs decorated Ag NPs.

***Characterization of O-CNT-PEG and O-CNT/Ag-PEG***

**Figure 2:**
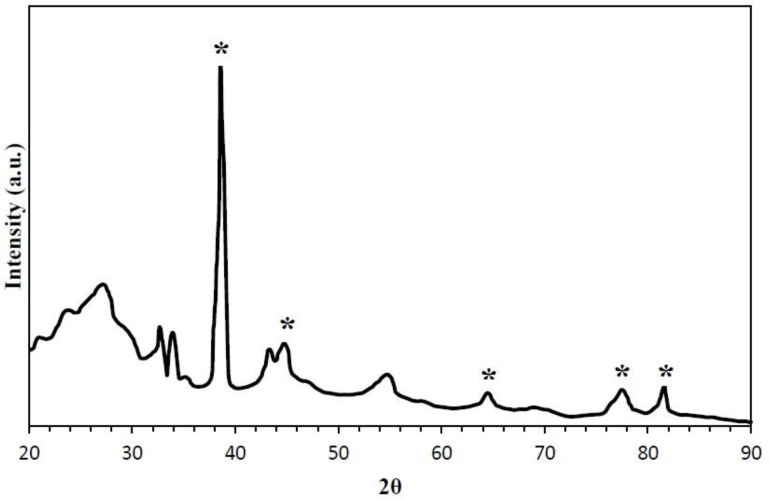
XRD pattern of synthesized O-CNT/Ag NPs (asterisk indicates the diffraction peaks of silver).

XRD pattern of synthesized O-CNT/Ag NP is given in Figure 2. Diffraction peaks of CNT and Ag NPs are clearly observed, which verifies the formation of Ag NPs in the presence of CNTs. This spectrum affirms the combination of both CNT and Ag NPs at 2θ values of 26, 38, 44, 52, 67, and 76 (28).

**Figure 3:**
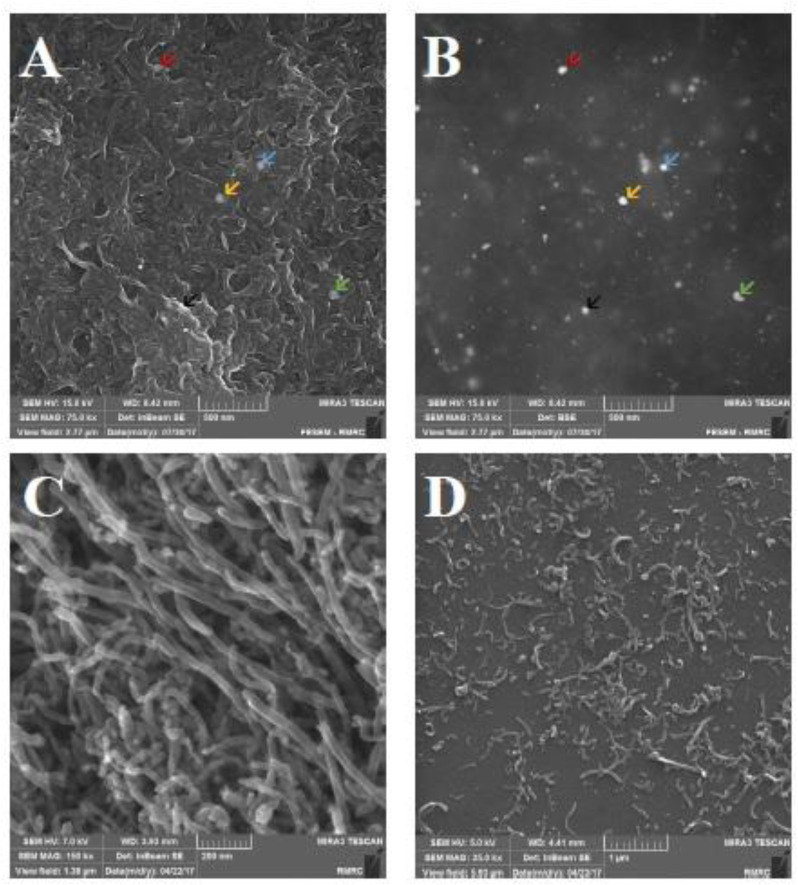
FESEM images of functionalized CNTs. (A) O-CNT/Ag-PEG image; based on inbeam secondray electron detector. (B) O-CNT/Ag-PEG image; based on back scatterd electron detector (arrows show silver NPs on O-CNTs in different technique of microscopy). (C) O-CNT image without any coating. (D) O-CNT-PEG image (PEGylation of O-CNTs).

**Figure 4:**
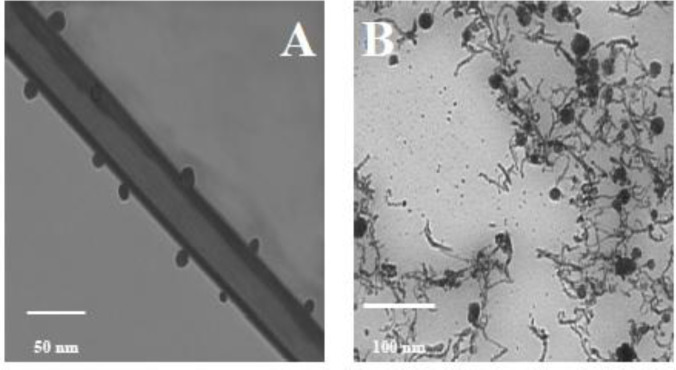
TEM images of modified CNTs. (A) TEM image of PEGylated O-CNTs. (B) TEM image of PEGylated O-CNT/Ag NPs

Morphological changes of O-CNT-PEG and O-CNT/Ag-PEG are shown in FESEM and TEM images (Figures 3 and 4). Silver nanospheres were stuck on O-CNTs in O-CNT/Ag-PEG NPs. Additionally, a thin layer surrounded O-CNTs and O-CNT/Ag NPs, which clearly established the presence of PEG on the surface of the NPs. It is observed that a continuous layer of PEG with an average thickness of 15 nm was formed on the surface of O-CNTs and O-CNT/Ag NPs. These results demonstrated that PEG chains were successfully wrapped on the NPs surfaces.

**Figure 5:**
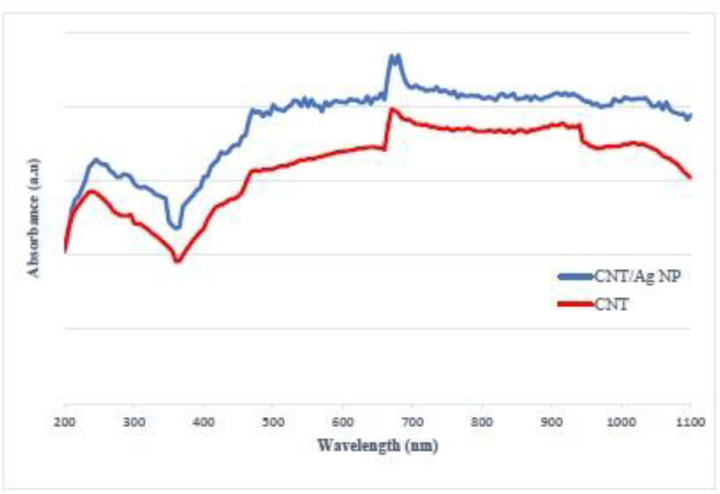
UV-Vis optical absorption spectrum of O-CNT-PEG (CNT) and O-CNT/Ag-PEG (CNT/Ag NP).

UV-Vis light absorption spectrum of O-CNT-PEG and O-CNT/Ag-PEG is displayed in Figure 5. According to this optical absorption curve, the maximum absorption wavelength of O-CNT-PEG and O-CNT/Ag-PEG was in the range of 670-940 nm. However, O-CNT/Ag-PEG NPs have a higher optical absorption than O-CNT-PEG due to silver presence in O-CNT/Ag-PEG. 

***Cell cytotoxicity of modified CNTs***

**Figure 6:**
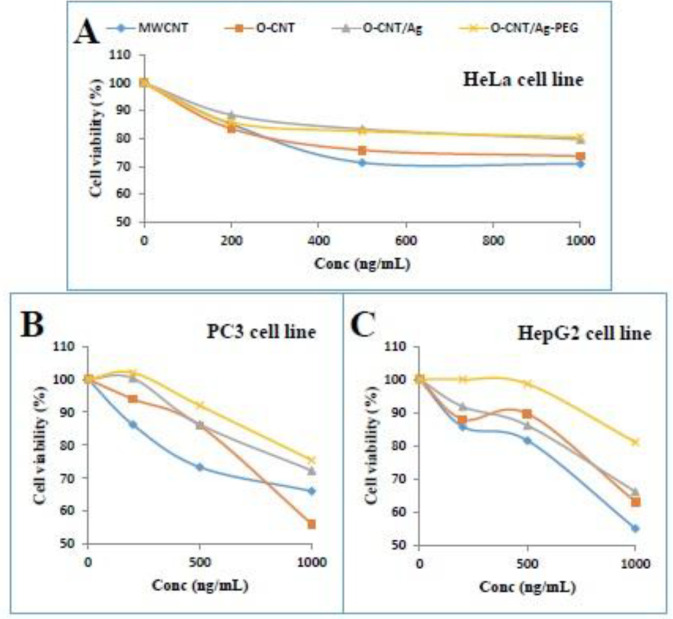
MTT assay of MWCNT, O-CNT, O-CNT/Ag, and O-CNT/Ag-PEG_1000_ against (A) HeLa, (B) PC3, and (C) HepG2 human cell lines

The cytotoxicity profile of MWCNT, O-CNT, O-CNT/Ag, and O-CNT/Ag-PEG against three human cell lines was evaluated to affirm the modification of O-CNT/Ag with PEG. HeLa, HepG2, and PC3 human cells were exposed to the mentioned NPs at different concentrations for 24 h and MTT assay was accomplished. Figures 6A-6C, demonstrate that at concentrations up to 1000 ng/mL, the toxicity of O-CNT/Ag-PEG NPs was relatively low in all three human cell lines. The presence of PEG around the O-CNT/Ag NPs enhanced the cell viability significantly. Coating of O-CNTs with silver and PEG increased the cell viability in HepG2, HeLa, and PC3 cell lines. 

***Hyperthermia therapy of tumors***

The *in-vivo *effects of O-CNT-PEG and O-CNT/Ag-PEG with laser excitation on the hypothermal implanted murine melanoma tumor were assessed by monitoring the tumor size.

**Figure 7:**
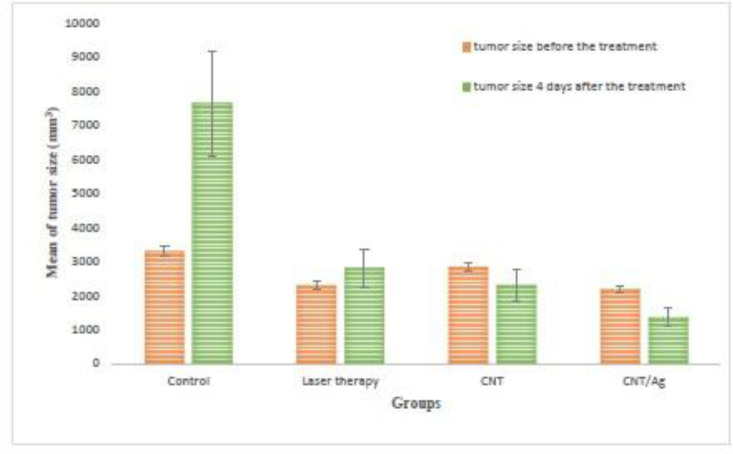
Tumor sizes of different groups before, and 4 days after the treatment with PTT procedure; (N = 5 in each group).

Tumor sizes were noted before and four days after laser incitation. Data were analyzed and a significant difference was seen between groups’ 1 and 2. The shrinking rate of tumor size in the CNT and CNT/Ag groups is obvious in Figure 7. As shown in this figure, the tumor size before and after the treatment was saved in each group. In the control group, it is obvious that the tumor growth was faster; however, in the CNT and CNT/Ag groups, the tumor size was decreased significantly. Using the mentioned NPs in combination with NIR laser irradiation, the tumor shrank to a small size while in the Control group some mice were expired before day four of the study. The tumor size increased at a slower rate in the Laser therapy group. This trend denoted that the average size of tumor before and four days after the treatment was increased in the Control group (from 3298.39 mm^3^ to 7636.927 mm^3^, respectively), but these values decreased in the CNT and CNT/Ag groups (from 2832.99 mm^3^ and 2180.722 mm^3^ to 2303.30 mm^3^ and 1351.516 mm^3^, respectively). The *P*-value was significantly different between whole groups after treatment.

**Figure 8:**
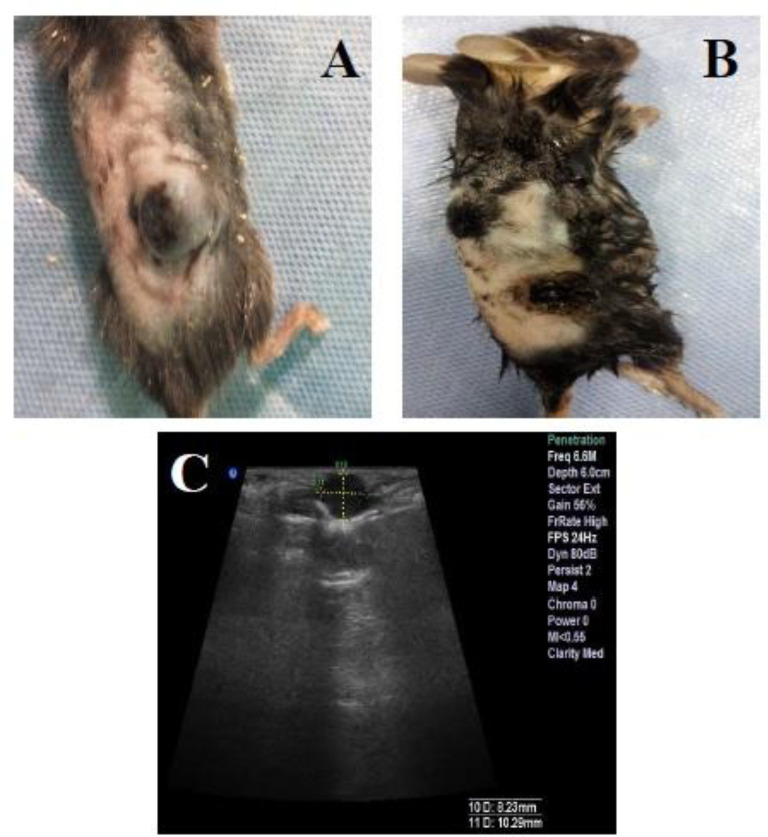
Stages of tumor treating in the CNT/Ag group with PTT procedure. (A and C) Photograph and ultrasonography image of a CNT/Ag case before the treatment, respectively. (B) Photograph of the mouse four days after the treatment (sonography was not possible four days after the treatment).

The slope of tumor size against time in the Control, Laser therapy, CNT, and CNT/Ag groups was approximately +1084.63 mm^3^/days, +130.19 mm^3^/days, –132.422 mm^3^/days, and –207.30 mm^3^/days, respectively (+ denotes increasing and – denotes decreasing of tumor size). The slope of tumor size reduction in the CNT and CNT/Ag groups is noticeable. Besides, ultrasound images were taken to determine the depth of tumors in all groups. The steps of tumor treatment in the CNT/Ag group are observable in Figure 8.

**Figure 9:**
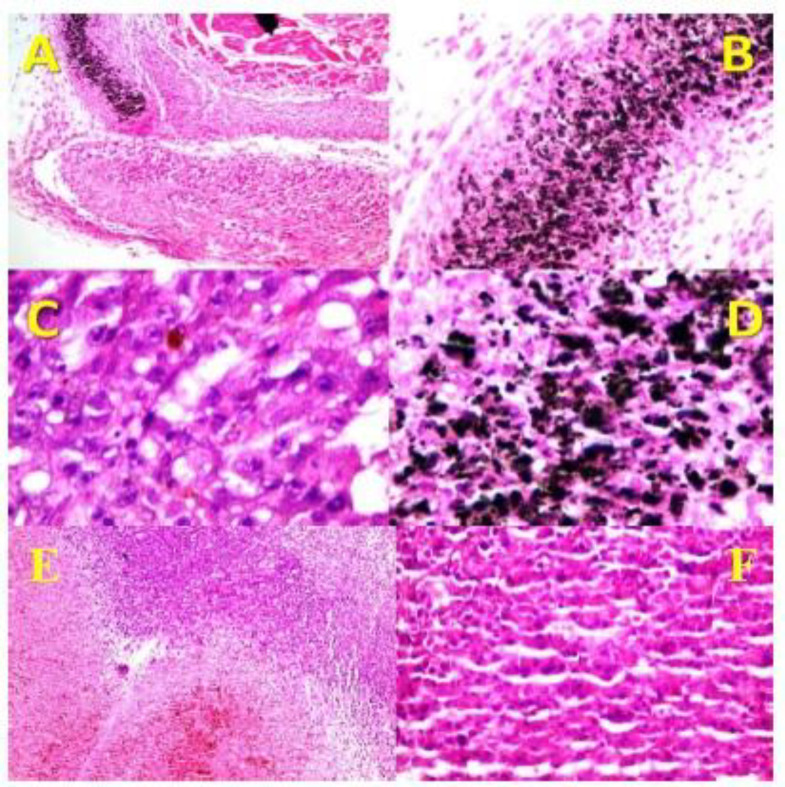
Histopathology of the skin of different groups. (A) It shows nodular melanoma associated with extensive necrosis in the CNT/Ag group (x40, H and E). (B and D) They show dense deposition of O-CNT/Ag-PEG_1000_ NPs in resolved area (B, x100, H&E) (D, x400, Hand E). (C) Areas of tumor residue next to resolved area with melanin pigment in the CNT/Ag group (x400, H and E). (E and F) They show nodular melanoma in the control group (E, x100, H and E) (F, x400, H and E).

Histopathologic examination was carried out for professional inspection. Gross evaluation of tumors showed severe shrinkage of tumor sizes in CNT and CNT/Ag groups compared to Laser therapy and Control groups. The microscopic evaluation demonstrated the attendance of nodular subtype malignant melanoma in all cases. Necrosis was found to be the most important discriminator between the CNT and CNT/Ag cases, and its percentage was very high in all cases allocated in the mentioned groups. As shown in Figure 9, in CNT and CNT/Ag groups, there was a direct association between the site of necrosis and deposition of NPs. Mitosis was higher in the control cases, and there was no evidence of ulceration, regressive fibrosis, vascular invasion, lymphocytic infiltration, neurotropism, and microsatellites in all cases. Table 1 shows the results in detail.

## Discussion

O-CNTs can absorb NIR light and transform its energy into heat impressively (5). In this research, we assessed the optical absorption of O-CNTs and O-CNTs decorated with Ag NPs in tumor depression by PTT procedure. To improve the dispersibility of CNTs in water, the hydrophilic polymer PEG was used to coat their surface. Based on different characterizations of these mentioned NPs, modification of CNTs with Ag NPs and PEG was established. Cytotoxicity study demonstrated that O-CNT/Ag was less cytotoxic than O-CNT, and O-CNT/Ag-PEG had the lowest toxicity among MWCNT, O-CNT, and O-CNT/Ag against HeLa, HepG2, and PC3 human cell lines. The cell viability after exposure to the mentioned NPs was significantly different (*p *< 0.05) in the mentioned three human cell lines. It is obvious that the sensitivity of various cell lines to the same NPs is different. NPs concentration and duration of exposure time to the NPs play an important role in toxicity evaluation as well. 

By injection of B16/F10 cell line, melanoma tumor was induced in mice. The effect of tumor destruction using O-CNT-PEG and O-CNT/Ag-PEG with the combination of laser excitation was assessed by irradiating the injected NPs in the tumor sites by an 808nm-2W CW NIR laser diode. Light penetration through the body in the NIR region is more convenient than in the visible spectrum (29, 30). Therefore, to incite injected NPs in the tumor site, a laser diode with a wavelength of 808 nm was chosen. The average size of the tumor in mice receiving O-CNT-PEG and O-CNT/Ag-PEG was reduced surprisingly in comparison with the other groups.

As-produced CNT has a hydrophobic surface and wants to bundle up. Hence, CNTs are insoluble in various solvents and biological media (31). The bundles of CNTs disturb the cell membrane of different cells and cause cell death. Besides, the attendance of residual metal catalysts in the CNTs causes them to show more toxic effects than the functionalized form of CNTs (5). Surfactants, nucleic acids, and polymers are usually used to functionalize CNTs (32). Two main procedures for functionalization of CNTs are noncovalent and covalent functionalization. In noncovalent functionalization, the electronic structure of CNTs is saved. Functionalization through this procedure is generally reproducible and uncomplicated (5). In this research, we generate dispersed CNTs by using PEG wrapping. After oxidation of pristine MWCNTs, carboxylic acid and hydroxyl groups formed at both sides of CNTs. Such functional groups could interact with PEG through hydrogenic bond, and ultimately form a fragile layer of polymer all around the CNTs. This hydrophilic polymer improves water dispersibility of CNTs (5, 6 and 33). Morphological changes of CNTs through PEGylation are obvious in FESEM and TEM images. The results of cytotoxicity assessment showed that pristine MWCNTs (as- produced) have more toxic effects on the three mentioned human cell lines than other modified MWCNTs. Aggregation of pristine MWCNTs and presence of metallic catalysts cause these toxic effects. Through oxidation of MWCNTs, metallic impurities are removed and, indeed, strong acid conditions cut the CNTs to shorter pieces and carboxylic functions are generated at the tips and around the side walls. O-CNTs have better dispersity than pristine CNTs and their cytotoxicity were decreased. After decorating Ag NPs on the side wall of O-CNTs, the cytotoxicity was not increased. By modification the hydrophobic surface of O-CNT/Ag by a layer of PEG, the cytotoxicity of these NPs against the cell lines was the lowest. Even with increasing the NPs concentration to 1000 ng/mL, about 80% of cells were alive (Figure 6).

These valuable results show that through simple surface modification of CNTs by a biocompatible and biodegradable polymer like PEG, we could reduce cytotoxicity of CNTs in different cell lines. Reduced cytotoxicity of CNTs through functionalization of their surfaces is demonstrated in the literature (34, 35).

In HeLa cells, we observed that when the cells were treated with high concentration (10000 ng/mL) of O-CNT/Ag-PEG and O-CNT/Ag, 67.13% and 55.63% of cells were alive after 24 h, respectively.

O-CNT/Ag-PEG NPs can be utilized to absorb NIR light and transform its energy into heat efficiently. These hybrid NPs can merge the attractive properties of CNTs and Ag NPs in optical fields. The produced heat can induce hyperthermia in cancerous cells due to their low heat tolerance in ratio to normal cells, and can ultimately cause necrosis in tumor sites. Therefore, localization and excitation of plasmonic NPs in tumor sites can be a potent procedure for treatment of solid tumors. Hashida *et al.* reported that the intra-tumoral injection of single-walled CNTs composite with a designed peptide, followed by NIR irradiation resulted in a rapid increase of the temperature to 43 °C in the subcutaneously inoculated colon 26 tumor and remarkable suppression of tumor growth compared with treatment by only NIR irradiation (36). They have reported that CNTs generate reactive oxygen species by NIR irradiation. As a result, both photodynamic and photothermal effects engage in destroying tumor cells (36, 37). 

Virani *et al.* employed functionalized single-walled CNTs with annexin V and phosphatidylserine for ablation of bladder cancer model via PTT. CNTs were administrated intravesically at a very low dose (0.1 mg per kg), followed 24 h later, mice were subjected to NIR light for only 30 sec exposure. The results showed the complete ablation of tumors and the absence of CNTs in the animal organism after 116 days (38).

Some reports established the photothermal effect of CNTs in the cell lines (37). Zhu *et al.* evaluated the potency of gold nanostars decorated MWCNTs against B16/F10 melanoma cells irradiated with an 808 nm laser (power density of 1.0 W cm^ -2 ^for 3 min). The MWCNTs/gold nanostars exhibited better effects in photothermal conversion and cancer cell ablation than gold nanostars alone and the gold nanospheres (39).

In this *in-vivo *study, O-CNT-PEG and O-CNT/Ag-PEG were injected into the melanoma tumor. After localization, these tumor regions were excited by a NIR laser diode. The rate of decrease in the tumor size of the CNT/Ag group was very rapid in comparison with the laser therapy group (Figure 7). Furthermore, histopathological assessment proved that efficient necrosis occurred in the CNT/Ag group (Figure 9). To achieve complete tumor cells ablation via the photothermal therapy method, it is necessary to optimize the protocol of NIR stimulation, such as repetition and duration of irradiation. 

**Table 1:** Results of histopathologic assessment

Groups	Necrosis (%)	Breslow’s thickness	Tumor stage after treatment according AJCC 2010
**CNT**	75	About 3mm	IIA
**CNT/Ag**	More than 95	About 3mm	IIA
**Laser therapy**	35	>4mm	IIB
**Control**	0	>4mm	IIB

## Conclusion

In this *in-vitro *and *in-vivo *study, a novel O-CNT/Ag-PEG NP was assessed to determine its destroying effect in the PTT procedure. Excitation of O-CNT/Ag-PEG with a laser diode induced heat generation and efficient damage to melanoma tumor *in-vivo*. Monitoring the mice after PTT for several months and using anti-cancer drugs are proposed for subsequent studies. According to this remarkable result, the next step is to assess the efficacy of O-CNT/Ag-PEG in tumor suppression by combining the PTT method with drug delivery method, which means loading anti-cancer agents on O-CNT/Ag-PEG NPs, and then irradiating the injected localized NPs in tumor sites through PTT technique.
